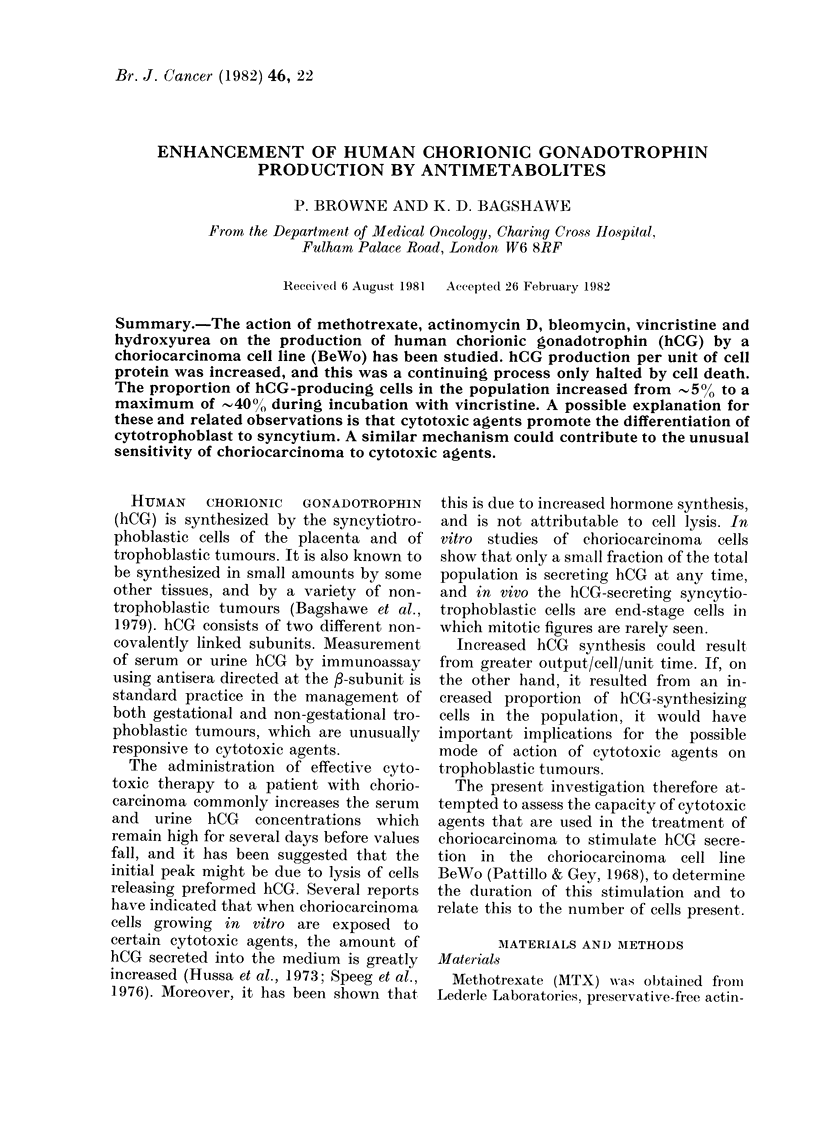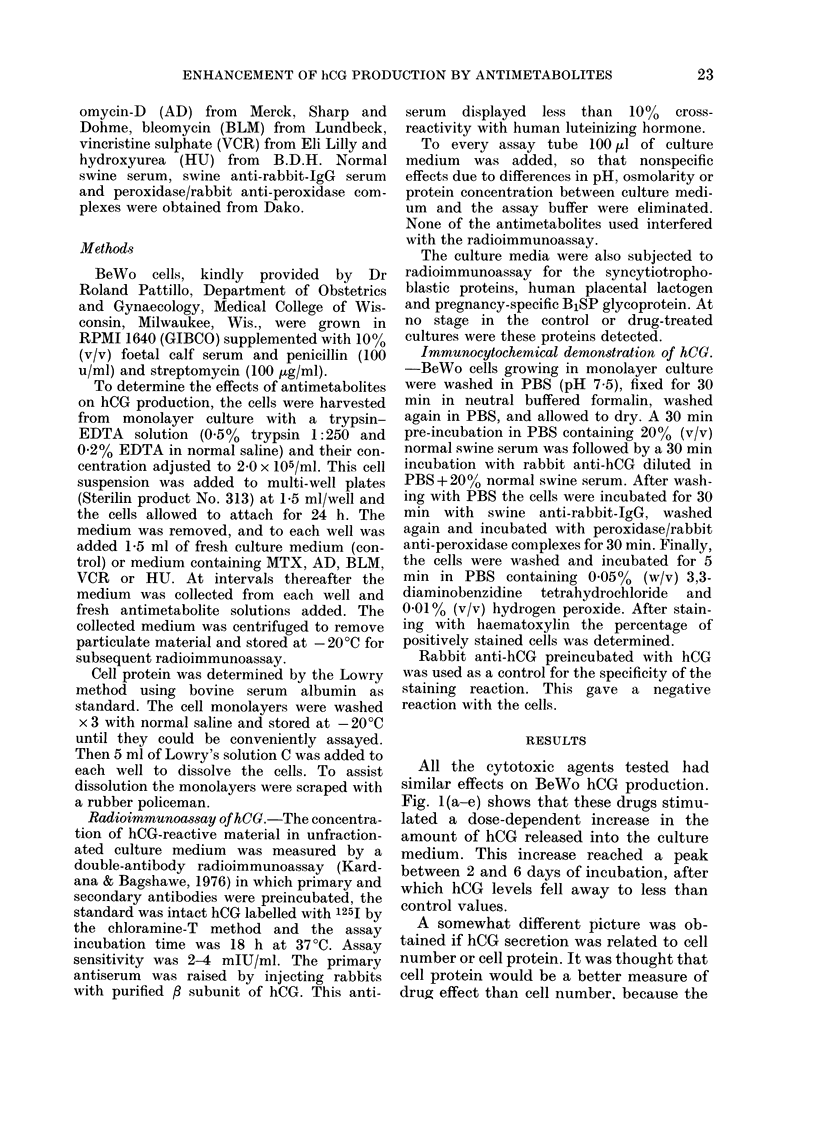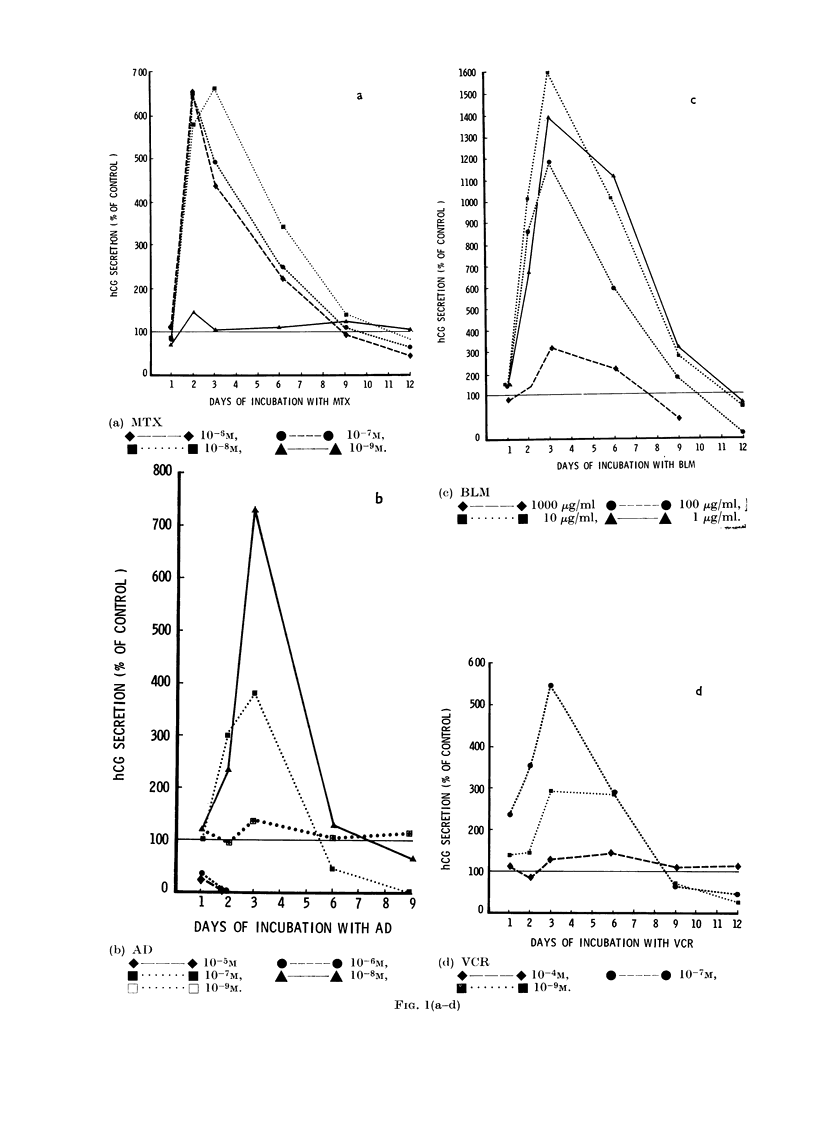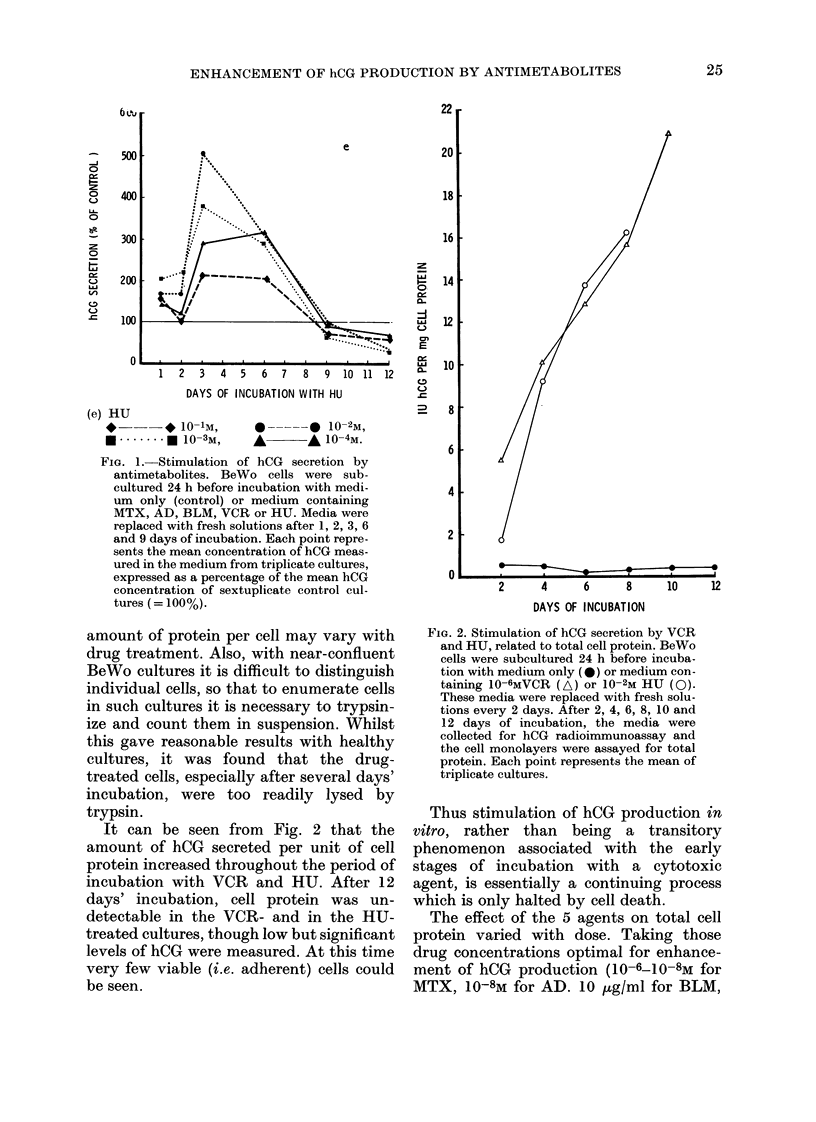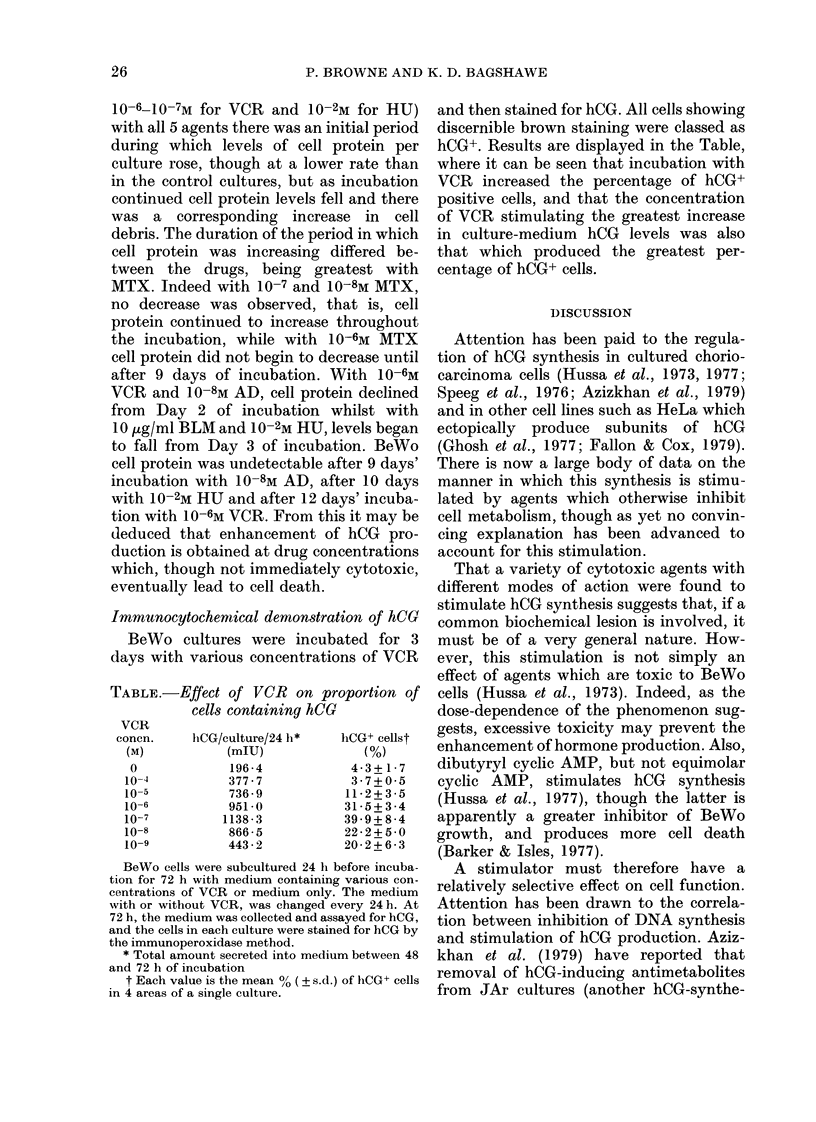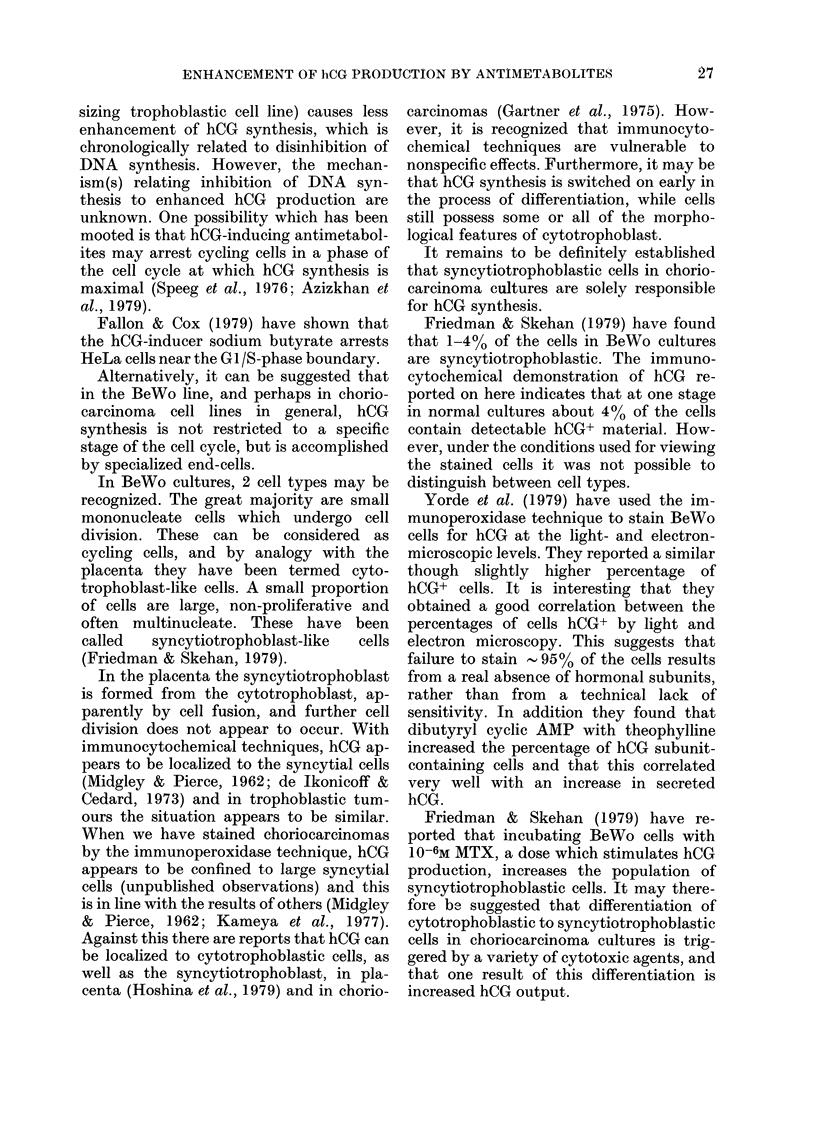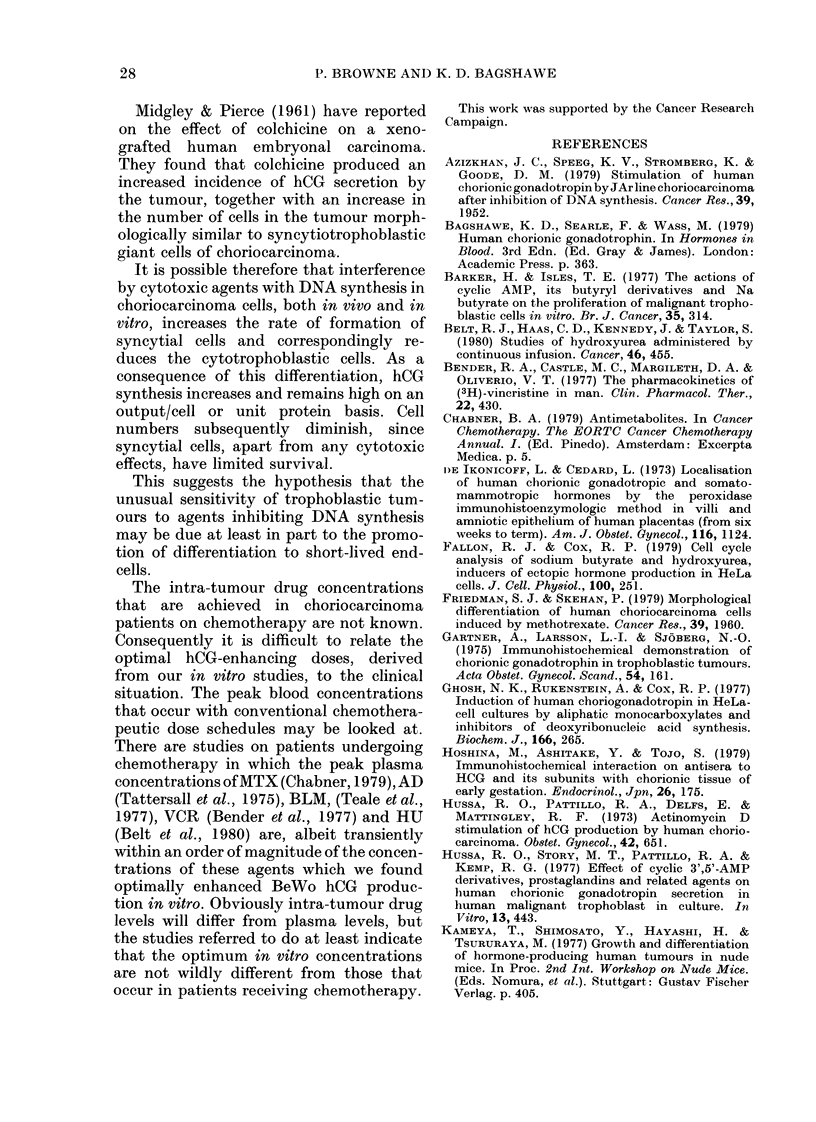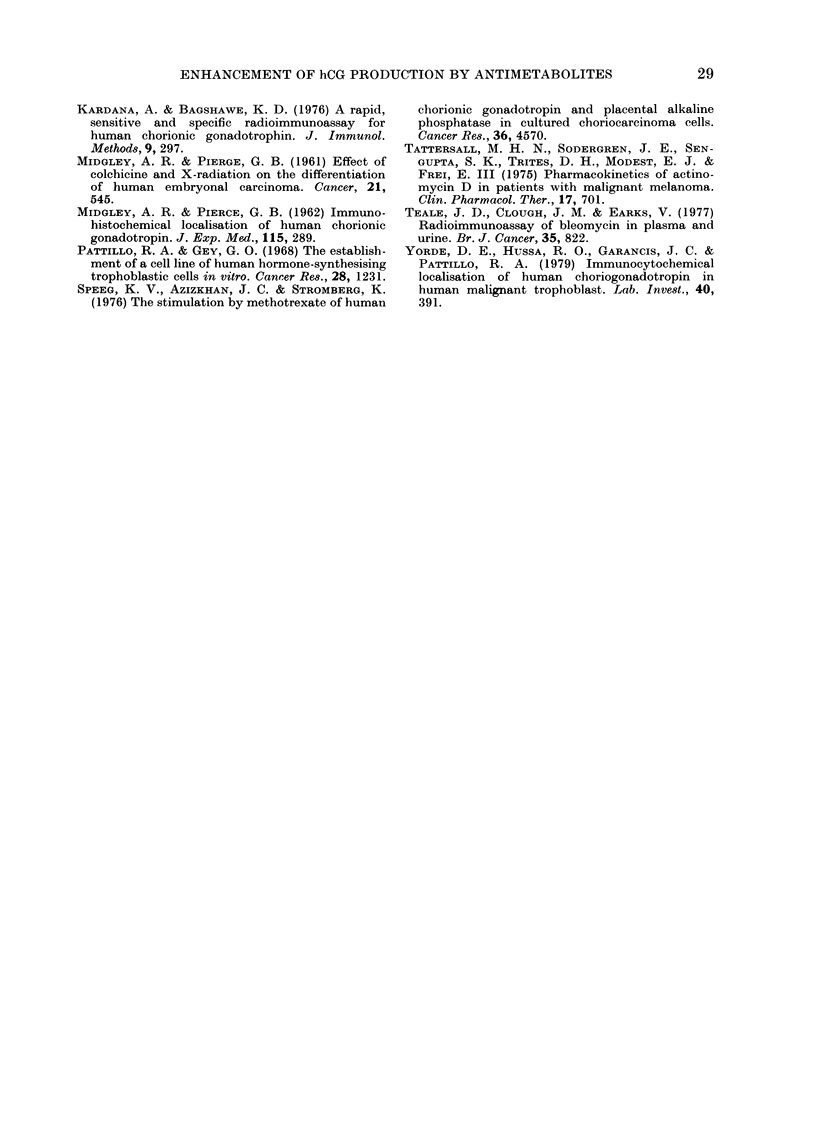# Enhancement of human chorionic gonadotrophin production by antimetabolites.

**DOI:** 10.1038/bjc.1982.160

**Published:** 1982-07

**Authors:** P. Browne, K. D. Bagshawe

## Abstract

The action of methotrexate, actinomycin D, bleomycin, vincristine and hydroxyurea on the production of human chorionic gonadotrophin (hCG) by a choriocarcinoma cell line (BeWo) has been studied. hCG production per unit of cell protein was increased, and this was a continuing process only halted by cell death. The proportion of hCG-producing cells in the population increased from approximately 5% to a maximum of approximately 40% during incubation with vincristine. A possible explanation for these and related observations is that cytotoxic agents promote the differentiation of cytotrophoblast to syncytium. A similar mechanism could contribute to the unusual sensitivity of choriocarcinoma to cytotoxic agents.


*Br. J. Cancer* (1982) **46**, 22

### ENHANCEMENT OF HUMAN CHORIONIC GONADOTROPHIN PRODUCTION BY ANTIMETABOLITES

P. BROWNE AND K. D. BAGSHAWE

*From the Department of Medical Oncology, Charing Cross Hospital, Fulham Palace Road, London W6 8RF*

Received 6 August 1981 Accepted 26 February 1982

**Summary.—The action of methotrexate, actinomycin D, bleomycin, vincristine and hydroxyurea on the production of human chorionic gonadotrophin (hCG) by a choriocarcinoma cell line (BeWo) has been studied. hCG production per unit of cell protein was increased, and this was a continuing process only halted by cell death. The proportion of hCG-producing cells in the population increased from ~5% to a maximum of ~40% during incubation with vincristine. A possible explanation for these and related observations is that cytotoxic agents promote the differentiation of cytotrophoblast to syncytium. A similar mechanism could contribute to the unusual sensitivity of choriocarcinoma to cytotoxic agents.**

HUMAN CHORIONIC GONADOTROPHIN (hCG) is synthesized by the syncytiotrophoblastic cells of the placenta and of trophoblastic tumours. It is also known to be synthesized in small amounts by some other tissues, and by a variety of non-trophoblastic tumours (Bagshawe *et al.*, 1979). hCG consists of two different non-covalently linked subunits. Measurement of serum or urine hCG by immunoassay using antisera directed at the $\beta$-subunit is standard practice in the management of both gestational and non-gestational trophoblastic tumours, which are unusually responsive to cytotoxic agents.

The administration of effective cytotoxic therapy to a patient with choriocarcinoma commonly increases the serum and urine hCG concentrations which remain high for several days before values fall, and it has been suggested that the initial peak might be due to lysis of cells releasing preformed hCG. Several reports have indicated that when choriocarcinoma cells growing *in vitro* are exposed to certain cytotoxic agents, the amount of hCG secreted into the medium is greatly increased (Hussa *et al.*, 1973; Speeg *et al.*, 1976). Moreover, it has been shown that this is due to increased hormone synthesis, and is not attributable to cell lysis. *In vitro* studies of choriocarcinoma cells show that only a small fraction of the total population is secreting hCG at any time, and *in vivo* the hCG-secreting syncytiotrophoblastic cells are end-stage cells in which mitotic figures are rarely seen.

Increased hCG synthesis could result from greater output/cell/unit time. If, on the other hand, it resulted from an increased proportion of hCG-synthesizing cells in the population, it would have important implications for the possible mode of action of cytotoxic agents on trophoblastic tumours.

The present investigation therefore attempted to assess the capacity of cytotoxic agents that are used in the treatment of choriocarcinoma to stimulate hCG secretion in the choriocarcinoma cell line BeWo (Pattillo & Gey, 1968), to determine the duration of this stimulation and to relate this to the number of cells present.

### MATERIALS AND METHODS

### *Materials*

Methotrexate (MTX) was obtained from Lederle Laboratories, preservative-free actinomycin-D (AD) from Merck, Sharp and Dohme, bleomycin (BLM) from Lundbeck, vincristine sulphate (VCR) from Eli Lilly and hydroxyurea (HU) from B.D.H. Normal swine serum, swine anti-rabbit-IgG serum and peroxidase/rabbit anti-peroxidase complexes were obtained from Dako.

### *Methods*

BeWo cells, kindly provided by Dr Roland Pattillo, Department of Obstetrics and Gynaecology, Medical College of Wisconsin, Milwaukee, Wis., were grown in RPMI 1640 (GIBCO) supplemented with 10% (v/v) foetal calf serum and penicillin (100 u/ml) and streptomycin (100 $\mu$g/ml).

To determine the effects of antimetabolites on hCG production, the cells were harvested from monolayer culture with a trypsin–EDTA solution (0·5% trypsin 1:250 and 0·2% EDTA in normal saline) and their concentration adjusted to $2{\cdot}0 \times 10^5$/ml. This cell suspension was added to multi-well plates (Sterilin product No. 313) at 1·5 ml/well and the cells allowed to attach for 24 h. The medium was removed, and to each well was added 1·5 ml of fresh culture medium (control) or medium containing MTX, AD, BLM, VCR or HU. At intervals thereafter the medium was collected from each well and fresh antimetabolite solutions added. The collected medium was centrifuged to remove particulate material and stored at $-20\,°C$ for subsequent radioimmunoassay.

Cell protein was determined by the Lowry method using bovine serum albumin as standard. The cell monolayers were washed $\times 3$ with normal saline and stored at $-20\,°C$ until they could be conveniently assayed. Then 5 ml of Lowry's solution C was added to each well to dissolve the cells. To assist dissolution the monolayers were scraped with a rubber policeman.

*Radioimmunoassay of hCG.*—The concentration of hCG-reactive material in unfractionated culture medium was measured by a double-antibody radioimmunoassay (Kardana & Bagshawe, 1976) in which primary and secondary antibodies were preincubated, the standard was intact hCG labelled with $^{125}I$ by the chloramine-T method and the assay incubation time was 18 h at 37 °C. Assay sensitivity was 2–4 mIU/ml. The primary antiserum was raised by injecting rabbits with purified $\beta$ subunit of hCG. This antiserum displayed less than 10% cross-reactivity with human luteinizing hormone.

To every assay tube 100 $\mu$l of culture medium was added, so that nonspecific effects due to differences in pH, osmolarity or protein concentration between culture medium and the assay buffer were eliminated. None of the antimetabolites used interfered with the radioimmunoassay.

The culture media were also subjected to radioimmunoassay for the syncytiotrophoblastic proteins, human placental lactogen and pregnancy-specific $B_1$SP glycoprotein. At no stage in the control or drug-treated cultures were these proteins detected.

*Immunocytochemical demonstration of hCG.*—BeWo cells growing in monolayer culture were washed in PBS (pH 7·5), fixed for 30 min in neutral buffered formalin, washed again in PBS, and allowed to dry. A 30 min pre-incubation in PBS containing 20% (v/v) normal swine serum was followed by a 30 min incubation with rabbit anti-hCG diluted in PBS + 20% normal swine serum. After washing with PBS the cells were incubated for 30 min with swine anti-rabbit-IgG, washed again and incubated with peroxidase/rabbit anti-peroxidase complexes for 30 min. Finally, the cells were washed and incubated for 5 min in PBS containing 0·05% (w/v) 3,3-diaminobenzidine tetrahydrochloride and 0·01% (v/v) hydrogen peroxide. After staining with haematoxylin the percentage of positively stained cells was determined.

Rabbit anti-hCG preincubated with hCG was used as a control for the specificity of the staining reaction. This gave a negative reaction with the cells.

### RESULTS

All the cytotoxic agents tested had similar effects on BeWo hCG production. Fig. 1(a–e) shows that these drugs stimulated a dose-dependent increase in the amount of hCG released into the culture medium. This increase reached a peak between 2 and 6 days of incubation, after which hCG levels fell away to less than control values.

A somewhat different picture was obtained if hCG secretion was related to cell number or cell protein. It was thought that cell protein would be a better measure of drug effect than cell number, because the

(a) MTX
◆———◆ $10^{-6}$M, ●- - - -● $10^{-7}$M,
■· · · · · ·■ $10^{-8}$M, ▲———▲ $10^{-9}$M.

(c) BLM
◆———◆ 1000 μg/ml ●- - - - -● 100 μg/ml,
■· · · · · ·■ 10 μg/ml, ▲———▲ 1 μg/ml.

(b) AD
◆———◆ $10^{-5}$M ●- - - - -● $10^{-6}$M,
■· · · · · ·■ $10^{-7}$M, ▲———▲ $10^{-8}$M,
□· · · · · ·□ $10^{-9}$M.

(d) VCR
◆———◆ $10^{-4}$M, ●- - - - -● $10^{-7}$M,
■· · · · · ·■ $10^{-9}$M.

Fig. 1(a–d)

(e) HU
◆———◆ $10^{-1}$M, ●-----● $10^{-2}$M,
■·······■ $10^{-3}$M, ▲———▲ $10^{-4}$M.

Fig. 1.—Stimulation of hCG secretion by antimetabolites. BeWo cells were subcultured 24 h before incubation with medium only (control) or medium containing MTX, AD, BLM, VCR or HU. Media were replaced with fresh solutions after 1, 2, 3, 6 and 9 days of incubation. Each point represents the mean concentration of hCG measured in the medium from triplicate cultures, expressed as a percentage of the mean hCG concentration of sextuplicate control cultures (= 100%).

Fig. 2. Stimulation of hCG secretion by VCR and HU, related to total cell protein. BeWo cells were subcultured 24 h before incubation with medium only (●) or medium containing $10^{-6}$MVCR (△) or $10^{-2}$M HU (○). These media were replaced with fresh solutions every 2 days. After 2, 4, 6, 8, 10 and 12 days of incubation, the media were collected for hCG radioimmunoassay and the cell monolayers were assayed for total protein. Each point represents the mean of triplicate cultures.

amount of protein per cell may vary with drug treatment. Also, with near-confluent BeWo cultures it is difficult to distinguish individual cells, so that to enumerate cells in such cultures it is necessary to trypsinize and count them in suspension. Whilst this gave reasonable results with healthy cultures, it was found that the drug-treated cells, especially after several days' incubation, were too readily lysed by trypsin.

It can be seen from Fig. 2 that the amount of hCG secreted per unit of cell protein increased throughout the period of incubation with VCR and HU. After 12 days' incubation, cell protein was undetectable in the VCR- and in the HU-treated cultures, though low but significant levels of hCG were measured. At this time very few viable (*i.e.* adherent) cells could be seen.

Thus stimulation of hCG production *in vitro*, rather than being a transitory phenomenon associated with the early stages of incubation with a cytotoxic agent, is essentially a continuing process which is only halted by cell death.

The effect of the 5 agents on total cell protein varied with dose. Taking those drug concentrations optimal for enhancement of hCG production ($10^{-6}$–$10^{-8}$M for MTX, $10^{-8}$M for AD. 10 $\mu$g/ml for BLM,

$10^{-6}$–$10^{-7}$M for VCR and $10^{-2}$M for HU) with all 5 agents there was an initial period during which levels of cell protein per culture rose, though at a lower rate than in the control cultures, but as incubation continued cell protein levels fell and there was a corresponding increase in cell debris. The duration of the period in which cell protein was increasing differed between the drugs, being greatest with MTX. Indeed with $10^{-7}$ and $10^{-8}$M MTX, no decrease was observed, that is, cell protein continued to increase throughout the incubation, while with $10^{-6}$M MTX cell protein did not begin to decrease until after 9 days of incubation. With $10^{-6}$M VCR and $10^{-8}$M AD, cell protein declined from Day 2 of incubation whilst with 10 $\mu$g/ml BLM and $10^{-2}$M HU, levels began to fall from Day 3 of incubation. BeWo cell protein was undetectable after 9 days' incubation with $10^{-8}$M AD, after 10 days with $10^{-2}$M HU and after 12 days' incubation with $10^{-6}$M VCR. From this it may be deduced that enhancement of hCG production is obtained at drug concentrations which, though not immediately cytotoxic, eventually lead to cell death.

*Immunocytochemical demonstration of hCG*

BeWo cultures were incubated for 3 days with various concentrations of VCR

TABLE.—*Effect of VCR on proportion of cells containing hCG*

| VCR concn. (M) | hCG/culture/24 h* (mIU) | $hCG^+$ cells† (%) |
|---|---|---|
| 0 | 196·4 | 4·3±1·7 |
| $10^{-4}$ | 377·7 | 3·7±0·5 |
| $10^{-5}$ | 736·9 | 11·2±3·5 |
| $10^{-6}$ | 951·0 | 31·5±3·4 |
| $10^{-7}$ | 1138·3 | 39·9±8·4 |
| $10^{-8}$ | 866·5 | 22·2±5·0 |
| $10^{-9}$ | 443·2 | 20·2±6·3 |

BeWo cells were subcultured 24 h before incubation for 72 h with medium containing various concentrations of VCR or medium only. The medium with or without VCR, was changed every 24 h. At 72 h, the medium was collected and assayed for hCG, and the cells in each culture were stained for hCG by the immunoperoxidase method.

* Total amount secreted into medium between 48 and 72 h of incubation

† Each value is the mean % (±s.d.) of $hCG^+$ cells in 4 areas of a single culture.

and then stained for hCG. All cells showing discernible brown staining were classed as $hCG^+$. Results are displayed in the Table, where it can be seen that incubation with VCR increased the percentage of $hCG^+$ positive cells, and that the concentration of VCR stimulating the greatest increase in culture-medium hCG levels was also that which produced the greatest percentage of $hCG^+$ cells.

### DISCUSSION

Attention has been paid to the regulation of hCG synthesis in cultured choriocarcinoma cells (Hussa *et al.*, 1973, 1977; Speeg *et al.*, 1976; Azizkhan *et al.*, 1979) and in other cell lines such as HeLa which ectopically produce subunits of hCG (Ghosh *et al.*, 1977; Fallon & Cox, 1979). There is now a large body of data on the manner in which this synthesis is stimulated by agents which otherwise inhibit cell metabolism, though as yet no convincing explanation has been advanced to account for this stimulation.

That a variety of cytotoxic agents with different modes of action were found to stimulate hCG synthesis suggests that, if a common biochemical lesion is involved, it must be of a very general nature. However, this stimulation is not simply an effect of agents which are toxic to BeWo cells (Hussa *et al.*, 1973). Indeed, as the dose-dependence of the phenomenon suggests, excessive toxicity may prevent the enhancement of hormone production. Also, dibutyryl cyclic AMP, but not equimolar cyclic AMP, stimulates hCG synthesis (Hussa *et al.*, 1977), though the latter is apparently a greater inhibitor of BeWo growth, and produces more cell death (Barker & Isles, 1977).

A stimulator must therefore have a relatively selective effect on cell function. Attention has been drawn to the correlation between inhibition of DNA synthesis and stimulation of hCG production. Azizkhan *et al.* (1979) have reported that removal of hCG-inducing antimetabolites from JAr cultures (another hCG-synthesizing trophoblastic cell line) causes less enhancement of hCG synthesis, which is chronologically related to disinhibition of DNA synthesis. However, the mechanism(s) relating inhibition of DNA synthesis to enhanced hCG production are unknown. One possibility which has been mooted is that hCG-inducing antimetabolites may arrest cycling cells in a phase of the cell cycle at which hCG synthesis is maximal (Speeg *et al.*, 1976; Azizkhan *et al.*, 1979).

Fallon & Cox (1979) have shown that the hCG-inducer sodium butyrate arrests HeLa cells near the G1/S-phase boundary.

Alternatively, it can be suggested that in the BeWo line, and perhaps in choriocarcinoma cell lines in general, hCG synthesis is not restricted to a specific stage of the cell cycle, but is accomplished by specialized end-cells.

In BeWo cultures, 2 cell types may be recognized. The great majority are small mononucleate cells which undergo cell division. These can be considered as cycling cells, and by analogy with the placenta they have been termed cytotrophoblast-like cells. A small proportion of cells are large, non-proliferative and often multinucleate. These have been called syncytiotrophoblast-like cells (Friedman & Skehan, 1979).

In the placenta the syncytiotrophoblast is formed from the cytotrophoblast, apparently by cell fusion, and further cell division does not appear to occur. With immunocytochemical techniques, hCG appears to be localized to the syncytial cells (Midgley & Pierce, 1962; de Ikonicoff & Cedard, 1973) and in trophoblastic tumours the situation appears to be similar. When we have stained choriocarcinomas by the immunoperoxidase technique, hCG appears to be confined to large syncytial cells (unpublished observations) and this is in line with the results of others (Midgley & Pierce, 1962; Kameya *et al.*, 1977). Against this there are reports that hCG can be localized to cytotrophoblastic cells, as well as the syncytiotrophoblast, in placenta (Hoshina *et al.*, 1979) and in choriocarcinomas (Gartner *et al.*, 1975). However, it is recognized that immunocytochemical techniques are vulnerable to nonspecific effects. Furthermore, it may be that hCG synthesis is switched on early in the process of differentiation, while cells still possess some or all of the morphological features of cytotrophoblast.

It remains to be definitely established that syncytiotrophoblastic cells in choriocarcinoma cultures are solely responsible for hCG synthesis.

Friedman & Skehan (1979) have found that 1–4% of the cells in BeWo cultures are syncytiotrophoblastic. The immunocytochemical demonstration of hCG reported on here indicates that at one stage in normal cultures about 4% of the cells contain detectable $hCG^{+}$ material. However, under the conditions used for viewing the stained cells it was not possible to distinguish between cell types.

Yorde *et al.* (1979) have used the immunoperoxidase technique to stain BeWo cells for hCG at the light- and electron-microscopic levels. They reported a similar though slightly higher percentage of $hCG^{+}$ cells. It is interesting that they obtained a good correlation between the percentages of cells $hCG^{+}$ by light and electron microscopy. This suggests that failure to stain $\sim 95\%$ of the cells results from a real absence of hormonal subunits, rather than from a technical lack of sensitivity. In addition they found that dibutyryl cyclic AMP with theophylline increased the percentage of hCG subunit-containing cells and that this correlated very well with an increase in secreted hCG.

Friedman & Skehan (1979) have reported that incubating BeWo cells with $10^{-6}$M MTX, a dose which stimulates hCG production, increases the population of syncytiotrophoblastic cells. It may therefore be suggested that differentiation of cytotrophoblastic to syncytiotrophoblastic cells in choriocarcinoma cultures is triggered by a variety of cytotoxic agents, and that one result of this differentiation is increased hCG output.

Midgley & Pierce (1961) have reported on the effect of colchicine on a xenografted human embryonal carcinoma. They found that colchicine produced an increased incidence of hCG secretion by the tumour, together with an increase in the number of cells in the tumour morphologically similar to syncytiotrophoblastic giant cells of choriocarcinoma.

It is possible therefore that interference by cytotoxic agents with DNA synthesis in choriocarcinoma cells, both *in vivo* and *in vitro*, increases the rate of formation of syncytial cells and correspondingly reduces the cytotrophoblastic cells. As a consequence of this differentiation, hCG synthesis increases and remains high on an output/cell or unit protein basis. Cell numbers subsequently diminish, since syncytial cells, apart from any cytotoxic effects, have limited survival.

This suggests the hypothesis that the unusual sensitivity of trophoblastic tumours to agents inhibiting DNA synthesis may be due at least in part to the promotion of differentiation to short-lived end-cells.

The intra-tumour drug concentrations that are achieved in choriocarcinoma patients on chemotherapy are not known. Consequently it is difficult to relate the optimal hCG-enhancing doses, derived from our *in vitro* studies, to the clinical situation. The peak blood concentrations that occur with conventional chemotherapeutic dose schedules may be looked at. There are studies on patients undergoing chemotherapy in which the peak plasma concentrations of MTX (Chabner, 1979), AD (Tattersall *et al.*, 1975), BLM, (Teale *et al.*, 1977), VCR (Bender *et al.*, 1977) and HU (Belt *et al.*, 1980) are, albeit transiently within an order of magnitude of the concentrations of these agents which we found optimally enhanced BeWo hCG production *in vitro*. Obviously intra-tumour drug levels will differ from plasma levels, but the studies referred to do at least indicate that the optimum *in vitro* concentrations are not wildly different from those that occur in patients receiving chemotherapy.

This work was supported by the Cancer Research Campaign.

REFERENCES

AZIZKHAN, J. C., SPEEG, K. V., STROMBERG, K. & GOODE, D. M. (1979) Stimulation of human chorionic gonadotropin by JAr line choriocarcinoma after inhibition of DNA synthesis. *Cancer Res.*, **39**, 1952.

BAGSHAWE, K. D., SEARLE, F. & WASS, M. (1979) Human chorionic gonadotrophin. In *Hormones in Blood*. 3rd Edn. (Ed. Gray & James). London: Academic Press. p. 363.

BARKER, H. & ISLES, T. E. (1977) The actions of cyclic AMP, its butyryl derivatives and Na butyrate on the proliferation of malignant trophoblastic cells *in vitro*. *Br. J. Cancer*, **35**, 314.

BELT, R. J., HAAS, C. D., KENNEDY, J. & TAYLOR, S. (1980) Studies of hydroxyurea administered by continuous infusion. *Cancer*, **46**, 455.

BENDER, R. A., CASTLE, M. C., MARGILETH, D. A. & OLIVERIO, V. T. (1977) The pharmacokinetics of ($^3$H)-vincristine in man. *Clin. Pharmacol. Ther.*, **22**, 430.

CHABNER, B. A. (1979) Antimetabolites. In *Cancer Chemotherapy. The EORTC Cancer Chemotherapy Annual. I.* (Ed. Pinedo). Amsterdam: Excerpta Medica. p. 5.

DE IKONICOFF, L. & CEDARD, L. (1973) Localisation of human chorionic gonadotropic and somatomammotropic hormones by the peroxidase immunohistoenzymologic method in villi and amniotic epithelium of human placentas (from six weeks to term). *Am. J. Obstet. Gynecol.*, **116**, 1124.

FALLON, R. J. & COX, R. P. (1979) Cell cycle analysis of sodium butyrate and hydroxyurea, inducers of ectopic hormone production in HeLa cells. *J. Cell. Physiol.*, **100**, 251.

FRIEDMAN, S. J. & SKEHAN, P. (1979) Morphological differentiation of human choriocarcinoma cells induced by methotrexate. *Cancer Res.*, **39**, 1960.

GARTNER, A., LARSSON, L.-I. & SJÖBERG, N.-O. (1975) Immunohistochemical demonstration of chorionic gonadotrophin in trophoblastic tumours. *Acta Obstet. Gynecol. Scand.*, **54**, 161.

GHOSH, N. K., RUKENSTEIN, A. & COX, R. P. (1977) Induction of human choriogonadotropin in HeLa-cell cultures by aliphatic monocarboxylates and inhibitors of deoxyribonucleic acid synthesis. *Biochem. J.*, **166**, 265.

HOSHINA, M., ASHITAKE, Y. & TOJO, S. (1979) Immunohistochemical interaction on antisera to HCG and its subunits with chorionic tissue of early gestation. *Endocrinol., Jpn*, **26**, 175.

HUSSA, R. O., PATTILLO, R. A., DELFS, E. & MATTINGLEY, R. F. (1973) Actinomycin D stimulation of hCG production by human choriocarcinoma. *Obstet. Gynecol.*, **42**, 651.

HUSSA, R. O., STORY, M. T., PATTILLO, R. A. & KEMP, R. G. (1977) Effect of cyclic 3',5'-AMP derivatives, prostaglandins and related agents on human chorionic gonadotropin secretion in human malignant trophoblast in culture. *In Vitro*, **13**, 443.

KAMEYA, T., SHIMOSATO, Y., HAYASHI, H. & TSURURAYA, M. (1977) Growth and differentiation of hormone-producing human tumours in nude mice. In Proc. *2nd Int. Workshop on Nude Mice.* (Eds. Nomura, *et al.*). Stuttgart: Gustav Fischer Verlag. p. 405.

KARDANA, A. & BAGSHAWE, K. D. (1976) A rapid, sensitive and specific radioimmunoassay for human chorionic gonadotrophin. *J. Immunol. Methods*, **9**, 297.

MIDGLEY, A. R. & PIERGE, G. B. (1961) Effect of colchicine and X-radiation on the differentiation of human embryonal carcinoma. *Cancer*, **21**, 545.

MIDGLEY, A. R. & PIERCE, G. B. (1962) Immunohistochemical localisation of human chorionic gonadotropin. *J. Exp. Med.*, **115**, 289.

PATTILLO, R. A. & GEY, G. O. (1968) The establishment of a cell line of human hormone-synthesising trophoblastic cells *in vitro*. *Cancer Res.*, **28**, 1231.

SPEEG, K. V., AZIZKHAN, J. C. & STROMBERG, K. (1976) The stimulation by methotrexate of human chorionic gonadotropin and placental alkaline phosphatase in cultured choriocarcinoma cells. *Cancer Res.*, **36**, 4570.

TATTERSALL, M. H. N., SODERGREN, J. E., SENGUPTA, S. K., TRITES, D. H., MODEST, E. J. & FREI, E. III (1975) Pharmacokinetics of actinomycin D in patients with malignant melanoma. *Clin. Pharmacol. Ther.*, **17**, 701.

TEALE, J. D., CLOUGH, J. M. & EARKS, V. (1977) Radioimmunoassay of bleomycin in plasma and urine. *Br. J. Cancer*, **35**, 822.

YORDE, D. E., HUSSA, R. O., GARANCIS, J. C. & PATTILLO, R. A. (1979) Immunocytochemical localisation of human choriogonadotropin in human malignant trophoblast. *Lab. Invest.*, **40**, 391.